# Transcriptome Analysis in Sheepgrass (*Leymus chinensis*): A Dominant Perennial Grass of the Eurasian Steppe

**DOI:** 10.1371/journal.pone.0067974

**Published:** 2013-07-04

**Authors:** Shuangyan Chen, Xin Huang, Xueqing Yan, Ye Liang, Yuezhu Wang, Xiaofeng Li, Xianjun Peng, Xingyong Ma, Lexin Zhang, Yueyue Cai, Tian Ma, Liqin Cheng, Dongmei Qi, Huajun Zheng, Xiaohan Yang, Xiaoxia Li, Gongshe Liu

**Affiliations:** 1 Key Laboratory of Plant Resources, Institute of Botany, the Chinese Academy of Sciences, Beijing, P. R. China; 2 Graduate Schoo1 of the Chinese Academy of Sciences, Beijing, P. R. China; 3 Shanghai-MOST Key Laboratory of Health and Disease Genomics, Chinese National Human Genome Center at Shanghai, Shanghai, P. R. China; 4 Biosciences Division, Oak Ridge National Laboratory, Oak Ridge, Tennessee, United States of America; Auburn University, United States of America

## Abstract

**Background:**

Sheepgrass [*Leymus chinensis* (Trin.) Tzvel.] is an important perennial forage grass across the Eurasian Steppe and is known for its adaptability to various environmental conditions. However, insufficient data resources in public databases for sheepgrass limited our understanding of the mechanism of environmental adaptations, gene discovery and molecular marker development.

**Results:**

The transcriptome of sheepgrass was sequenced using Roche 454 pyrosequencing technology. We assembled 952,328 high-quality reads into 87,214 unigenes, including 32,416 contigs and 54,798 singletons. There were 15,450 contigs over 500 bp in length. BLAST searches of our database against Swiss-Prot and NCBI non-redundant protein sequences (nr) databases resulted in the annotation of 54,584 (62.6%) of the unigenes. Gene Ontology (GO) analysis assigned 89,129 GO term annotations for 17,463 unigenes. We identified 11,675 core Poaceae-specific and 12,811 putative sheepgrass-specific unigenes by BLAST searches against all plant genome and transcriptome databases. A total of 2,979 specific freezing-responsive unigenes were found from this RNAseq dataset. We identified 3,818 EST-SSRs in 3,597 unigenes, and some SSRs contained unigenes that were also candidates for freezing-response genes. Characterizations of nucleotide repeats and dominant motifs of SSRs in sheepgrass were also performed. Similarity and phylogenetic analysis indicated that sheepgrass is closely related to barley and wheat.

**Conclusions:**

This research has greatly enriched sheepgrass transcriptome resources. The identified stress-related genes will help us to decipher the genetic basis of the environmental and ecological adaptations of this species and will be used to improve wheat and barley crops through hybridization or genetic transformation. The EST-SSRs reported here will be a valuable resource for future gene-phenotype studies and for the molecular breeding of sheepgrass and other Poaceae species.

## Background

*Leymus* is a genus of the grass family (Triticeae; Poaceae). Approximately 34 species of *Leymus* have been identified, and they are widely distributed along the coast of the North Sea, central Asia, eastern Asia, Alaska, and western North America [1]. In addition to being used for forage and conservation, *Leymus* is one of several perennial Triticeae genera used in wide-hybridization wheat breeding [2]. Several *Leymus* species have been successfully hybridized with wheat, and some of the resulting introgression lines display potentially useful traits, including salt tolerance [3], resistance to *Fusarium* head blight [4], [5], and biological nitrification inhibition [6].

Sheepgrass [*Leymus chinensis* (Trin.) Tzvel.] is an important perennial grass species in the *Leymus* genus. It is one of the typical grassland communities in the Eurasian steppe region, and it is widely distributed on the eastern Eurasian steppe, including the outer Baikal area of Russia, western North Korea, Mongolia, the Northeast Plain, the Northern Plain, and the Inner Mongolian Plateau of China [7]. The total area of sheepgrass grasslands in Asia is approximately 420,000 km^2^, of which 220,000 km^2^ are located in China. These grasslands play important roles in soil and water conservation, ecological construction and support of livestock farming, especially in northern China [8].

The significant role of sheepgrass in environmental protection is due to its thick and long belowground rhizomes, with many adventitious roots at each node. It is a self-incompatible species, which often enforces outcrossing and may have increased the geographic distribution of genetic diversity [9]. Sheepgrass has diverse environmental adaptations and can grow across diverse soil and climate conditions. It can endure the extremely low temperature of −47.5°C, survive drought when soil moisture might be less than 6% during dry seasons, and grow well at a concentration of 600 mmol/L of NaC1 and 175 mmol/L of Na_2_CO_3_ [10], [11]. Its high vegetative productivity, high protein content, and good palatability also make this species an important forage grass for animal husbandry [12].

Because of its important role in environmental protection, many researchers from a macro perspective have paid attention to how sheepgrass responds to global changes such as high temperature, drought, and CO_2_ doubling [13]–[17]. However, little attention has been paid to deciphering the genetic basis of its environmental adaptations, largely due to the limited genomic resources in sheepgrass. Thus far, only 1,815 ESTs and 51 protein sequences from sheepgrass have been deposited in public databases [18]. Gene discovery is also lagging, and only a few genes have been cloned and functionally validated [19], [20].

The advent of high-throughput next generation sequencing (NGS) technologies, such as Roche/454, Illumina/Solexa and ABI/SOLiD, has made it possible to generate genome resources at a large scale and relatively low cost [21], [22]. However, whole-genome sequencing is currently expensive and impractical for sheepgrass, which has a very large genome (9.65 Gb for a haploid genome). NGS technologies have been effectively used to generate large-scale transcriptome data in several plant species, such as *Medicago* [23], *Arabidopsis* [24], maize [25], barley [26], soybean [27], chickpea [28], [29], and developing oilseeds [30]. Recently, a transcriptome data was generated in sheepgrass (*Leymus chinensis*) under saline-alkaline treatment using Roche-454 massive parallel pyrosequencing technology and a large number of saline-alkaline responsive differentially expressed genes (DEGs) were obtained [31]. In this study, in order to further enrich sheepgrass transcriptome resources, obtain freezing stress resistance genes, accelerate our understanding of the genetic basis of stress tolerance traits, and discover useful genes and molecular marker for the future molecular breeding of sheepgrass and other Poaceae crops, such as wheat and barley, we generate a large collection of ESTs using 454 pyrosequencing technologies and present a comprehensive transcriptome characterization of sheepgrass, including assessments of transcriptome assembly, annotation, gene family and functional representation, useful gene discovery, SSR identification, and a phylogenic analysis of sheepgrass compared to other Poaceae species.

## Results

### Generation and *de novo* Assembly of Sheepgrass Transcriptome Data

**Figure 1:**
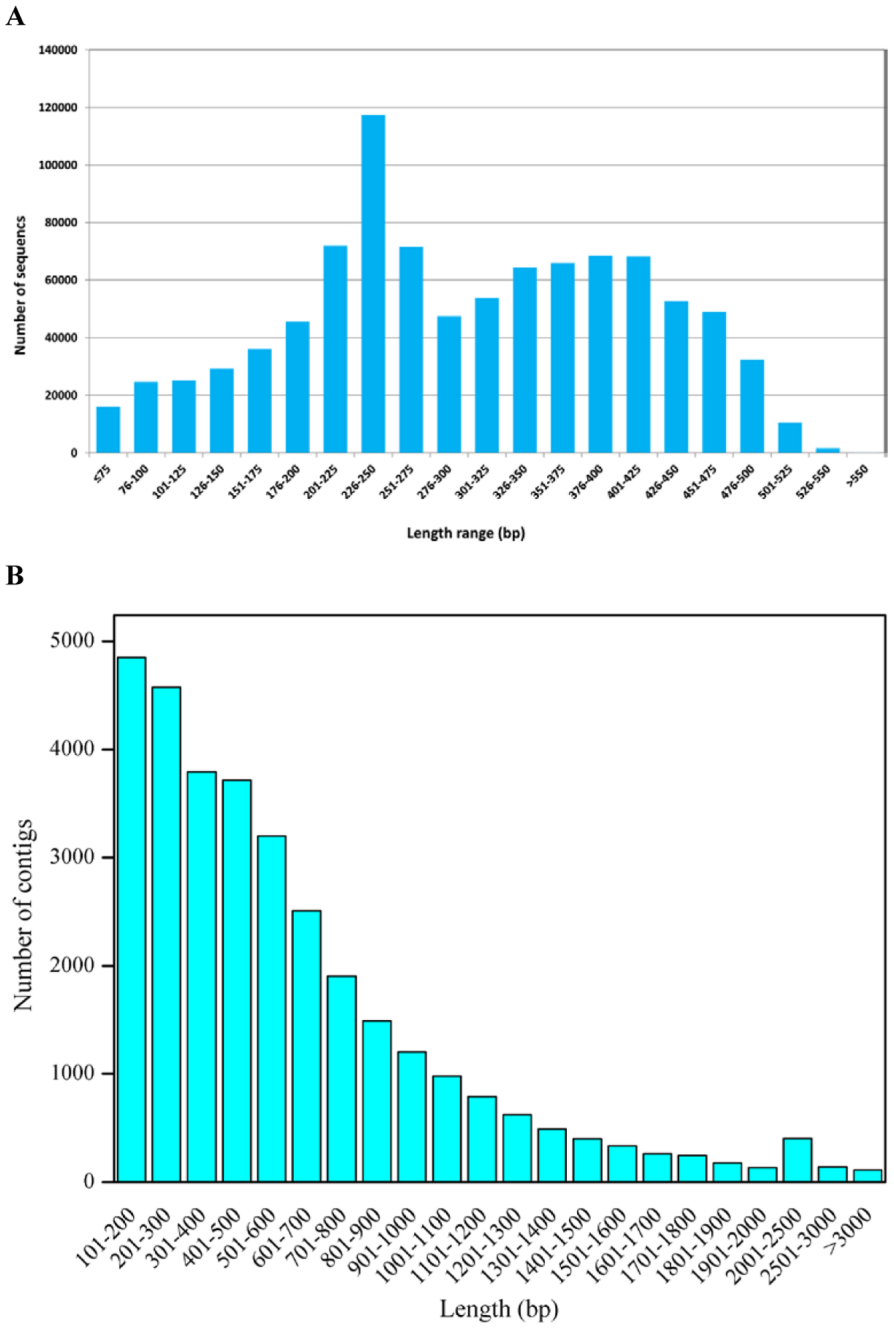
Length distribution of reads and contigs. (A) Reads length distribution. (B) Length distribution of contigs bigger than 100 bp.

In this study, we performed long-read transcriptome sequencing of five libraries from different tissue or treatment samples in sheepgrass via a GS FLX sequencer. More than 1 million reads of Q20 quality were obtained. Reads were passed through several quality control filters. After removing low-quality reads (Phred quality score of <20), short reads (<60 bp) and reads belonging to mitochondria and plastids, a total of 952,328 high-quality reads corresponding to mRNAs with an average length of 300 bp were obtained. The number of high-quality reads for different tissue samples ranged from 128,981 to 350,322. These reads covered a total of 285,531,328 bases (Table 1). The length distribution of these high-quality reads is shown in Figure 1A.

**Table 1:** Summary of 454 sequencing data generated for sheepgrass transcriptome and quality filtering.

Library/tissue type	Total reads^1^	Low-quality reads^2^	Removed reads^3^	High-quality mRNA reads^4^	Average length (bp) 5
Mixed	420,178	60,112	9,744	350,322	249
Buds (−40°C)	139,466	5,417	884	133,165	348
Buds (−15°C)	207,500	10,598	1,541	195,361	335
Buds (25°C)	152,005	6,535	971	144,499	347
Spikes	132,401	2,782	638	128,981	282
Total	1,051,550	85,444	13,778	952,328	300

1 Total number of reads separated for each libray/tissue type.

2 Number of low-quality reads (Phred quality score of <20) removed.

3 Number of short reads (<60 bp) and reads belonging to mitochondria and plastids.

4 Number of high-quality reads corresponding to mRNA sequences used for further analysis.

5 Average length of high-quality mRNA reads.

**Figure 2:**
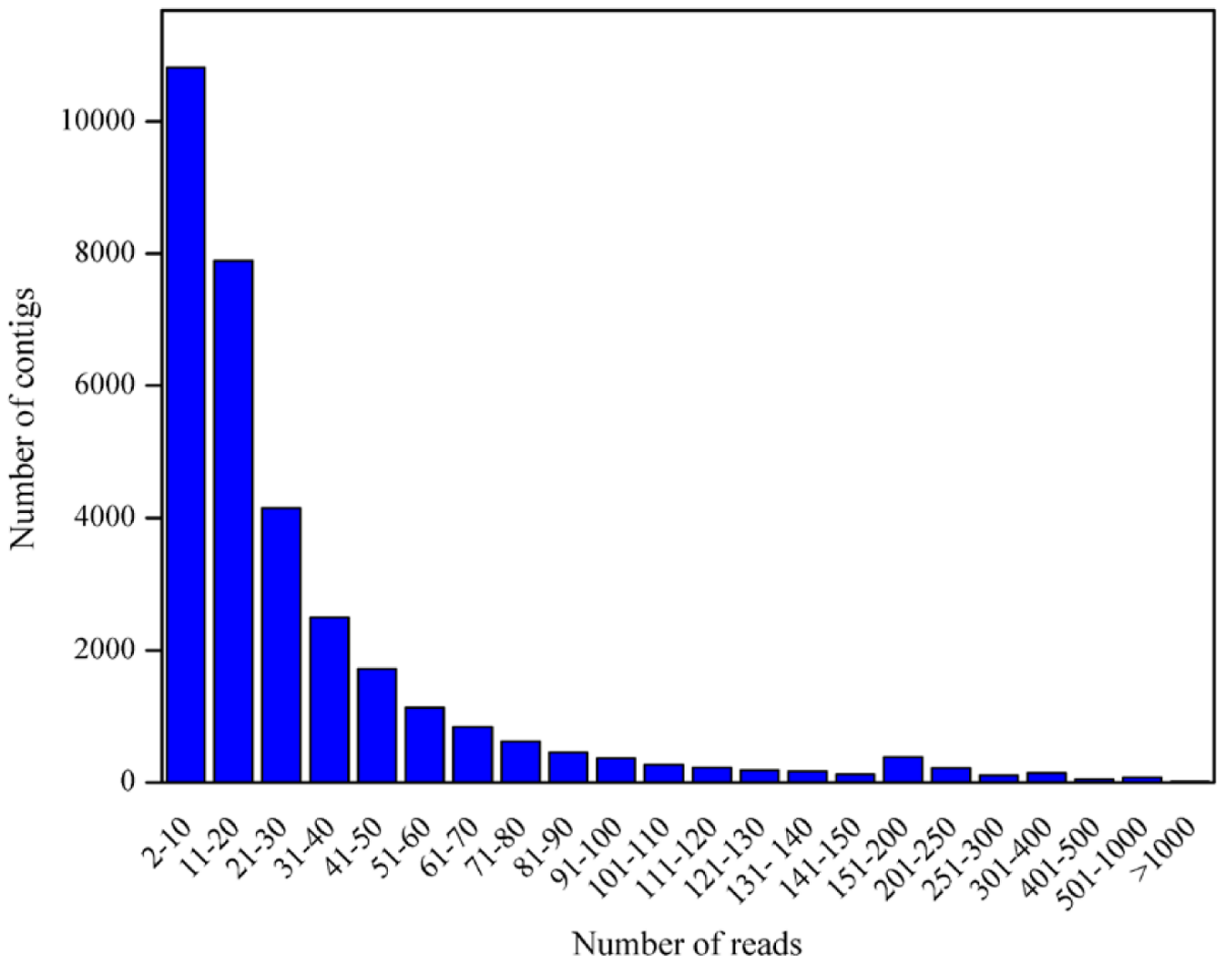
Histogram of the average read-depth coverage for assembled contigs.

**Figure 3:**
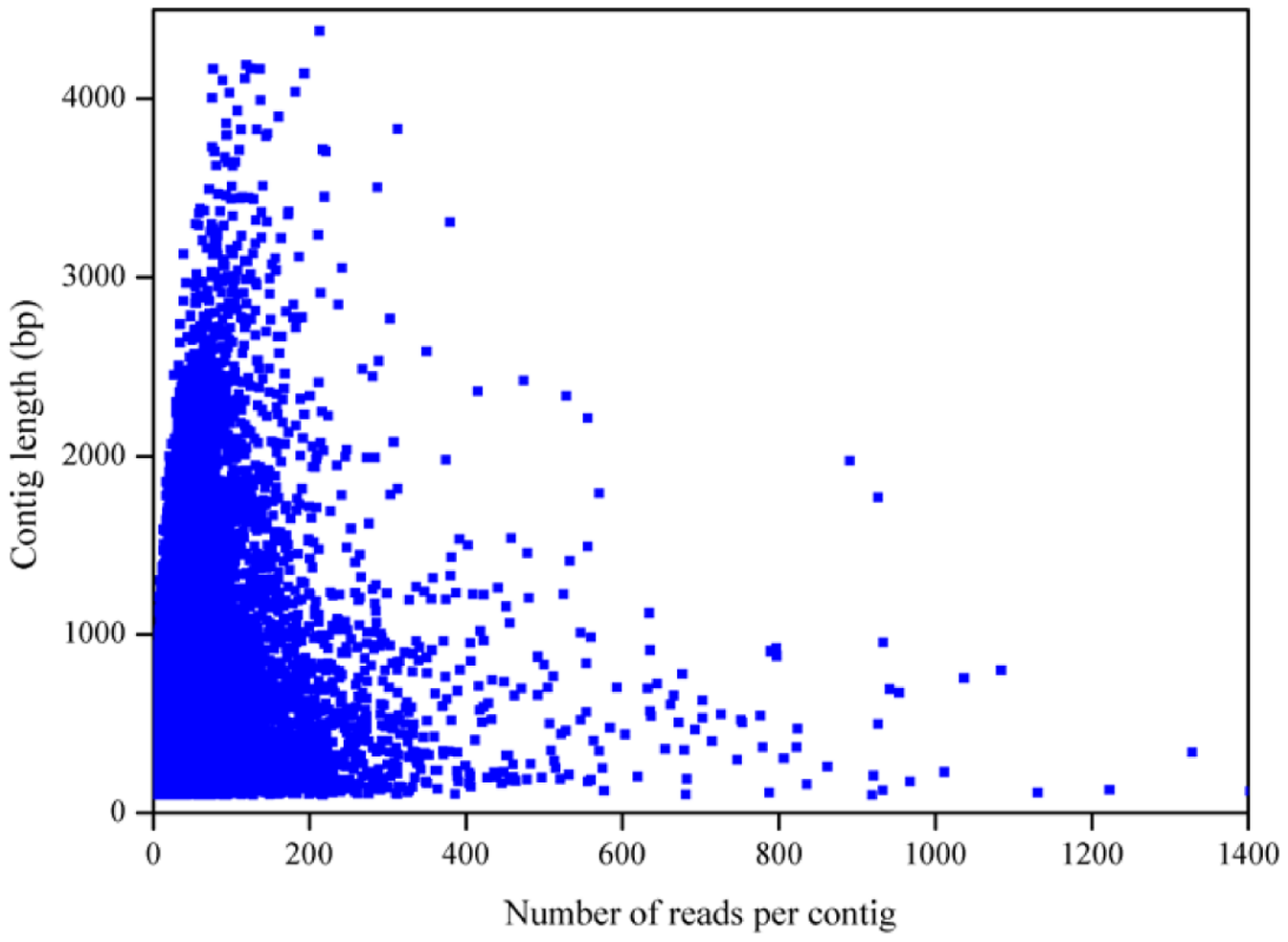
Scatter plot representing the number of reads per contig for each contig length.

Using the Newbler 2.5 (pl) assembly program, we generated a total of 87,214 unigenes, including 32,416 contigs (≥100 bp) and 54,798 singletons (≥300 bp) (Table 2). The length distribution of contigs bigger than 100 bp is shown in Figure 1B. The mean contig size and N50 were 607 bp and 813 bp, respectively. About half of the contigs (15,450; 48%) were equal or greater than 500 bp in length, and the mean contig size was 960 bp with an N50 of 1,019 bp. Among contigs bigger than 500 bp, approximately 42–44% of the contigs showed ≥80% coverage of *Brachypodium distachyon* and rice proteins by BLASTX against the genome of these two species at a cutoff E-value of 1e-5. There were 5,110 contigs ≥1,000 bp. The largest contig was 6,588 bp in length (Table 2). The contig coverage ranges from 2 to more than 1,000 reads per contig, with the majority of contigs covered by less than 30 reads (Figure 2). There is a positive relationship between the length of a contig and the number of reads it contains (Figures 2 and 3).

**Table 2:** Newbler 2.5 assembly statistics of sheepgrass transcripts.

Parameter	Numbers/Percentage (%)
Number of contigs	32,416
Total bases of contigs (bp)	19,691,750
Number of singletons ≥100 bp	118860
Number of singletons ≥300 bp	54,798
Total bases of singletons ≥300 bp	21,448,067
Contig mean length (bp)	607
Contig N50 length (bp)	813
Number of contig ≥500 bp	15,450
Mean length of contig ≥500 bp	960
N50 length of contig ≥500 bp	1,019
Contigs ≥500 bp with significant hits (%) 1	14418 (93.3%)
Contigs ≥500 bp with ≥80% coverage 2	6869 (44.5%)
Contigs ≥500 bp with significant hits (%) 3	14,321 (92.7%)
Contigs ≥500 bp with ≥80% coverage 4	6540 (42.3%)
Large contigs ≥1000 bp	5,110
Max length (bp)	6,588

1 Contigs showing significant hits (E-value≤1e-5) with *Brachypodium distachyon* proteins.

2 Contigs showing ≥80% coverage of *Brachypodium distachyon* proteins.

3 Contigs showing significant hits (E-value≤1e-5) with rice proteins.

4 Contigs showing ≥80% coverage of rice proteins.

To assess potential contamination in this assembly, all assembled unique sequences were compared to microbial proteins present in the Refseq databases at NCBI using the BLASTX algorithm at an E-value threshold of 1×10^−7^. These analyses showed that the contig and singleton sequences displayed a match of 0.02% and 0.09%, respectively, to microbial sequences, which indicated that most of the assembled sequences were from sheepgrass.

### Functional Annotation and Profile Description

To annotate the assembled unigenes, the predicted proteins of 87,214 unigenes generated by Newbler 2.5 (pl) were subjected to BLASTX searches against public protein databases using an E threshold of 1e-5 and protein identity no less than 30%. Overall, 54584 (62.6%) unigenes had homologous sequences in the Swiss-Prot and NCBI non-redundant protein sequences (nr) databases.

**Figure 4:**
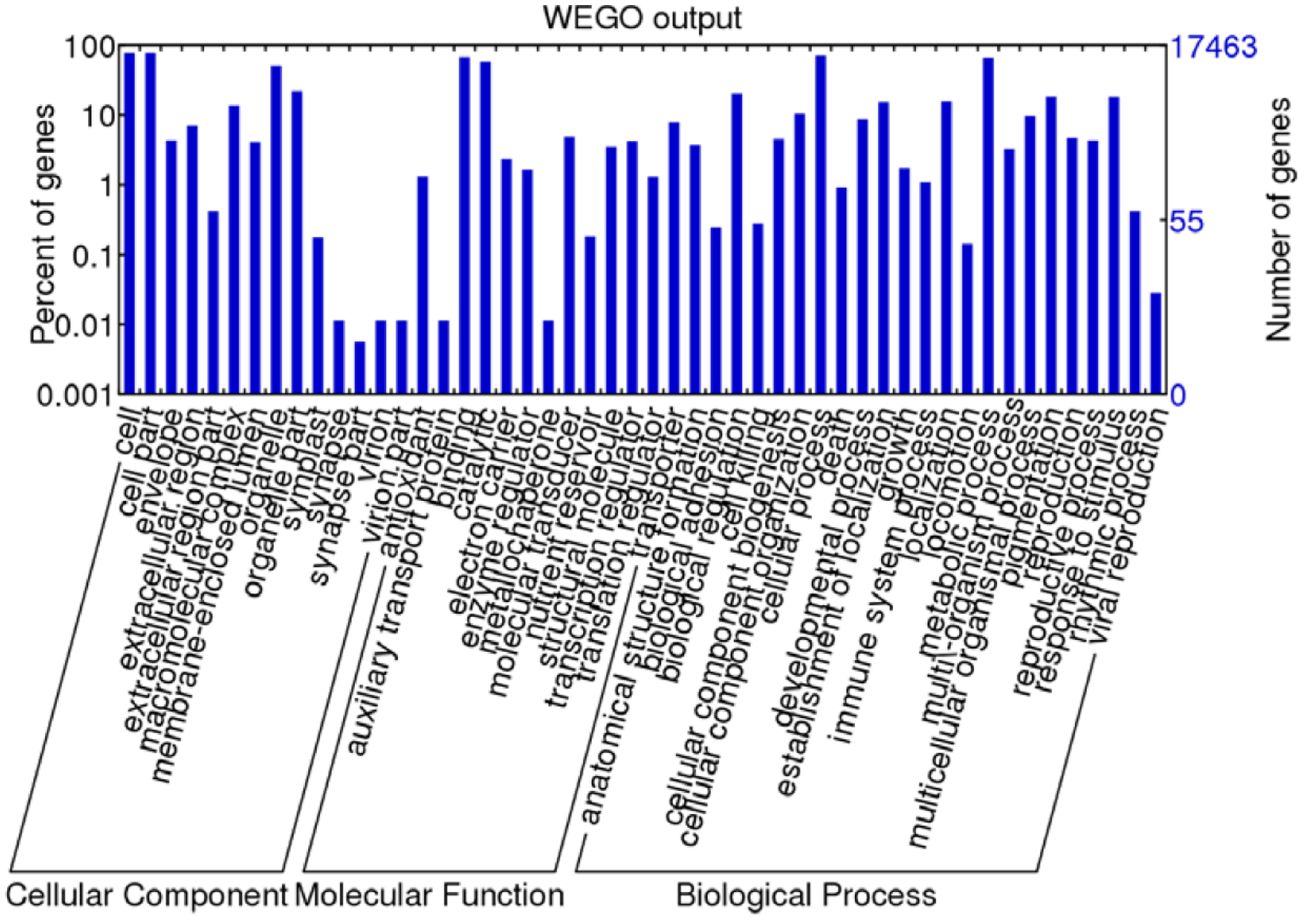
Histogram presentation of Gene Ontology classification. Venn diagram of the distribution of plant GO terms associated with sheepgrass unigenes represented in biological process, cellular component and molecular function categories.

The unigenes were further annotated with Gene Ontology (GO) terms. A total of 17,463 unigenes were assigned 89,129 GO term annotations, which could be classified into 3 categories: biological process, molecular function, and cellular component. Within the biological process category, the terms “cellular process”, “metabolic process”, “response to stimulus”, “biological regulation”, and “pigmentation” were dominant. In the cellular component category, most unigenes were assigned to “cell”, “cell part”, and “organelle”. In the molecular function category, the major GO terms were “binding”, “catalytic activity”, and “transporter” (Figure 4).

**Figure 5:**
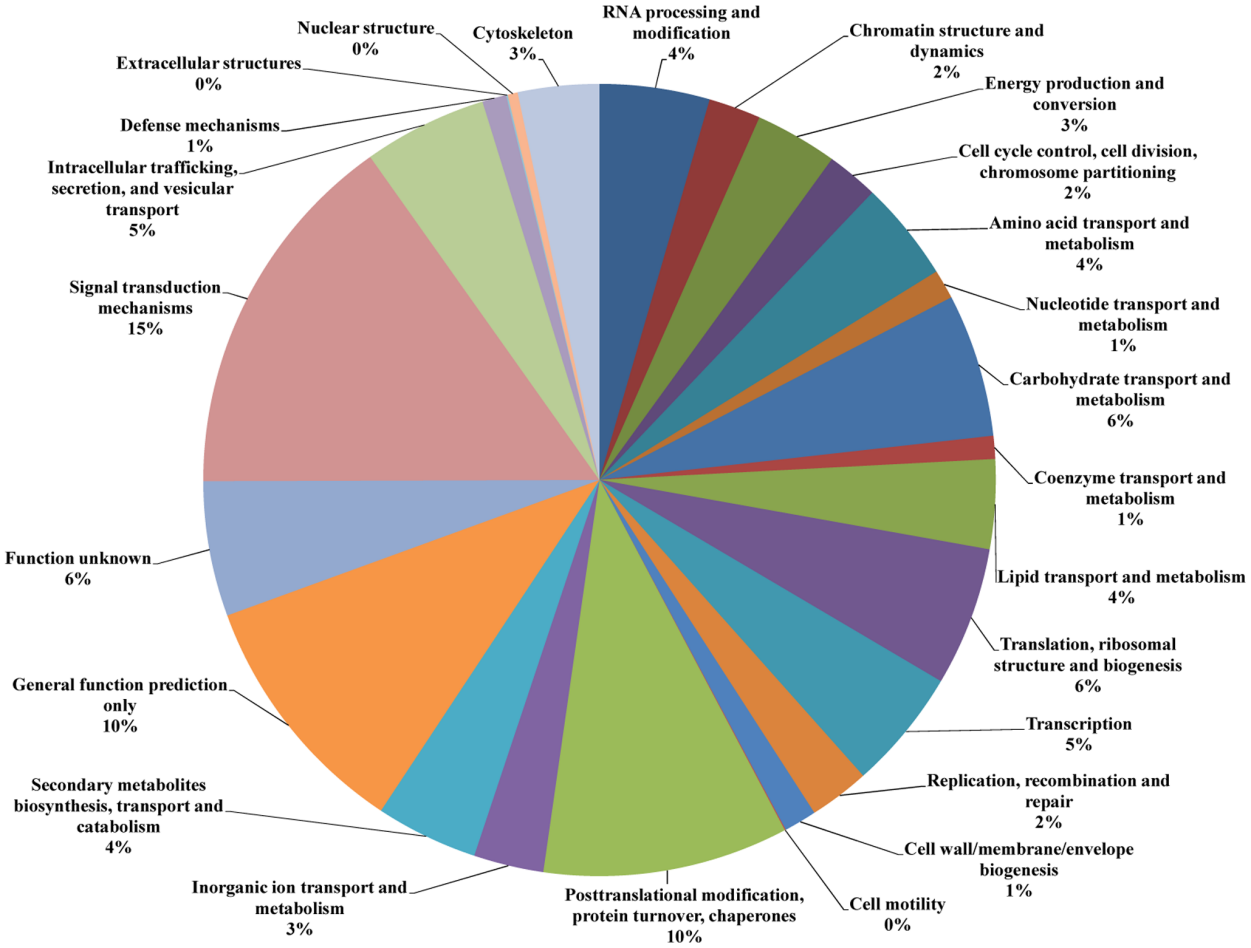
KOG function classification. All unigenes were aligned to the KOG database to predict and categorize possible functions. A total of 25,319 unigenes were assigned to 25 classifications.

All unigenes were further annotated and classified based on EuKaryotic Orthologous Groups (KOG) category. A total of 25,319 unigenes were assigned functional annotations and grouped into 25 functional categories (Figure 5). Within these categories, “signal transduction mechanisms” (15%), “posttranslational modification, protein turnover, chaperones” (10%), and “general function prediction only” (10%) were dominant, followed by carbohydrate transport and metabolism (6%), and “translation, ribosomal structure and biogenesis” (6%). Among “signal transduction mechanisms”, the most abundant type of unigene in this category was protein kinases. In addition, 6% of the unigenes belonged to the “function unknown” category.

**Figure 6:**
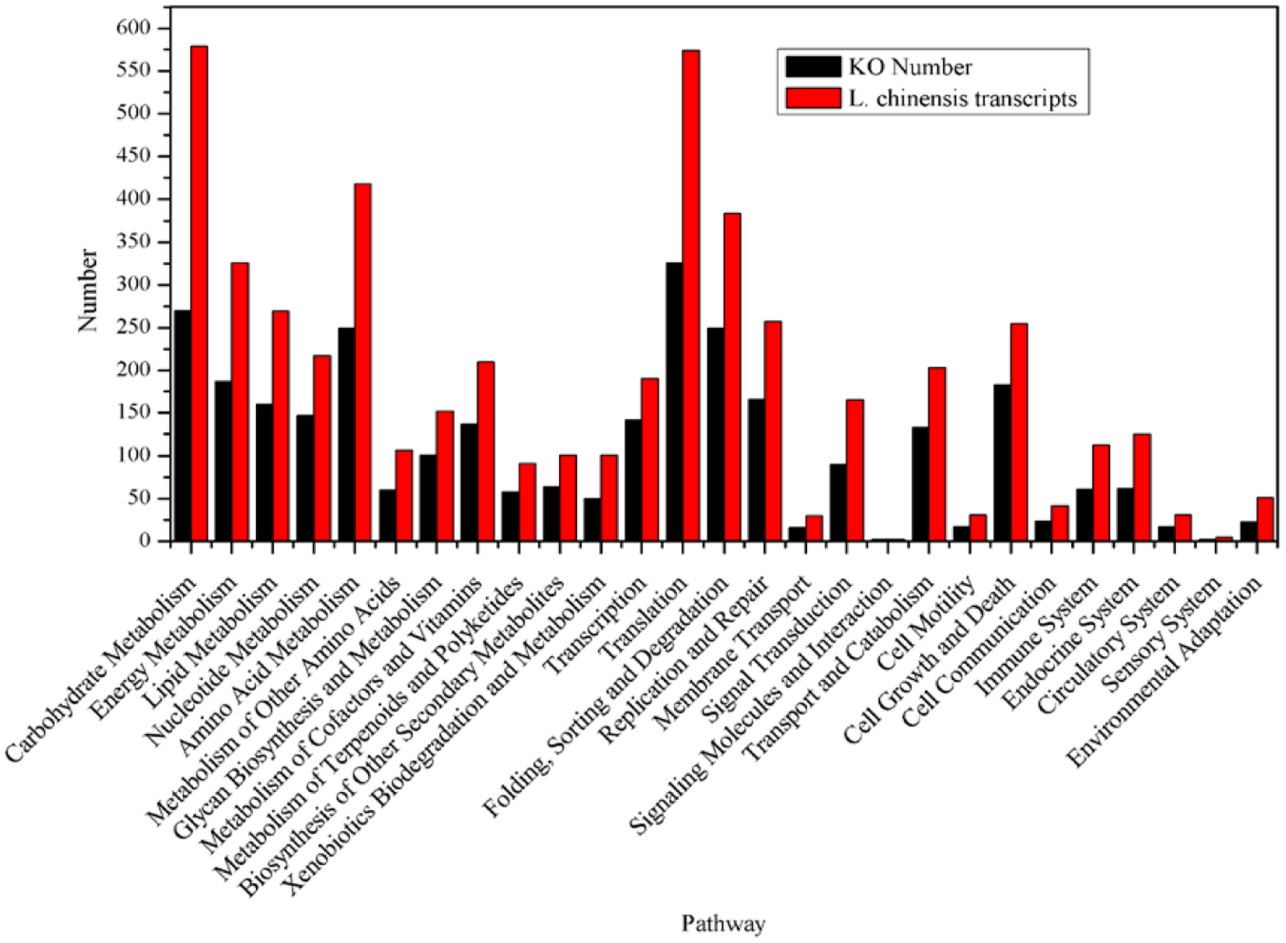
Histogram presentation of KEGG classification. The y-axis indicates the number of unigenes assigned to a specific pathway. The x-axis indicates the KEGG pathway.

The Kyoto Encyclopedia of Genes and Genomes (KEGG) pathway analysis revealed that diverse pathways were represented in our transcriptome dataset. Among these pathways, “carbohydrate metabolism”, “amino acid metabolism”, “energy metabolism”, “translation”, and “folding, sorting and degradation” were 5 most represented pathways (Figure 6). “Ribosome”, “spliceosome”, “RNA transport”, “purine metabolism” and “oxidative phosphorylation” were the 5 most represented subclass pathways. Some important pathways involved in signal transduction were also identified, including “MAPK signaling pathway”, “calcium signaling pathway” and “plant hormone signal transduction” (Table S1).

**Figure 7:**
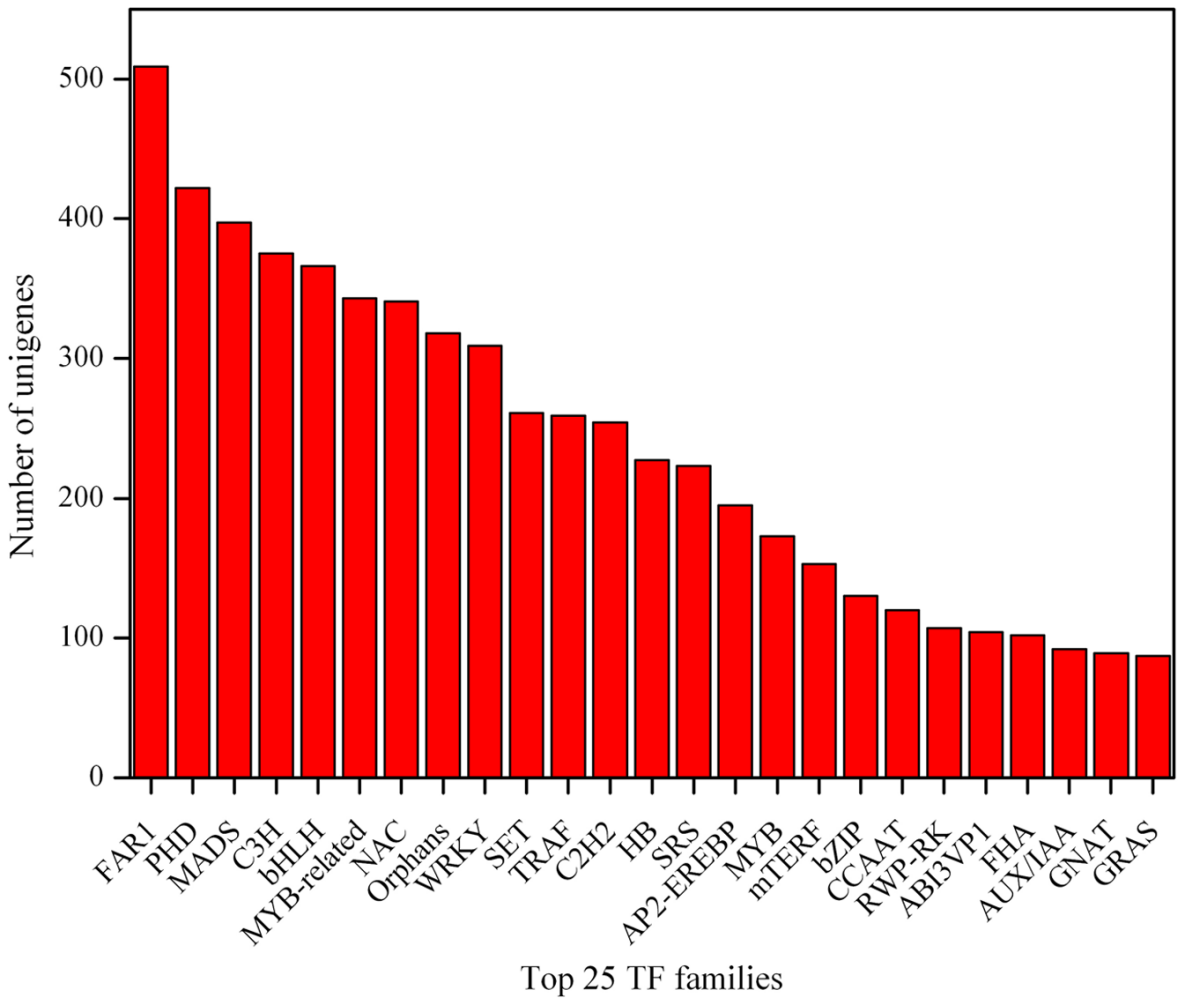
Top 25 transcription factor families. The y-axis indicates the number of unigenes assigned to a specific TF family. The x-axis indicates the top 25 TF families.

We also analyzed transcription factors (TFs) with all unigenes by BLASTX searches against the Plant Transcription Factor Database (version 3.0) (E-value ≤1e-10). A total of 7,223 unigenes were identified as encoding for TFs belonging to 78 families. The largest TF family was FAR1, which contained 509 unigenes. The next largest families were PHD, MADS, C3H, bHLH, MYB-related, NAC, and WRKY-family TFs. The top 25 TF families in the sheepgrass unigenes are shown in Figure 7. Among these, WRKY, AP2-EREBP and bZIP transcription factors play significant roles in responses to biotic and abiotic stresses. Many of the genes of the MADS family are involved in different steps of flower development.

### Core Poaceae-specific Unigenes and Putatively Novel Sheepgrass Unigenes

**Figure 8:**
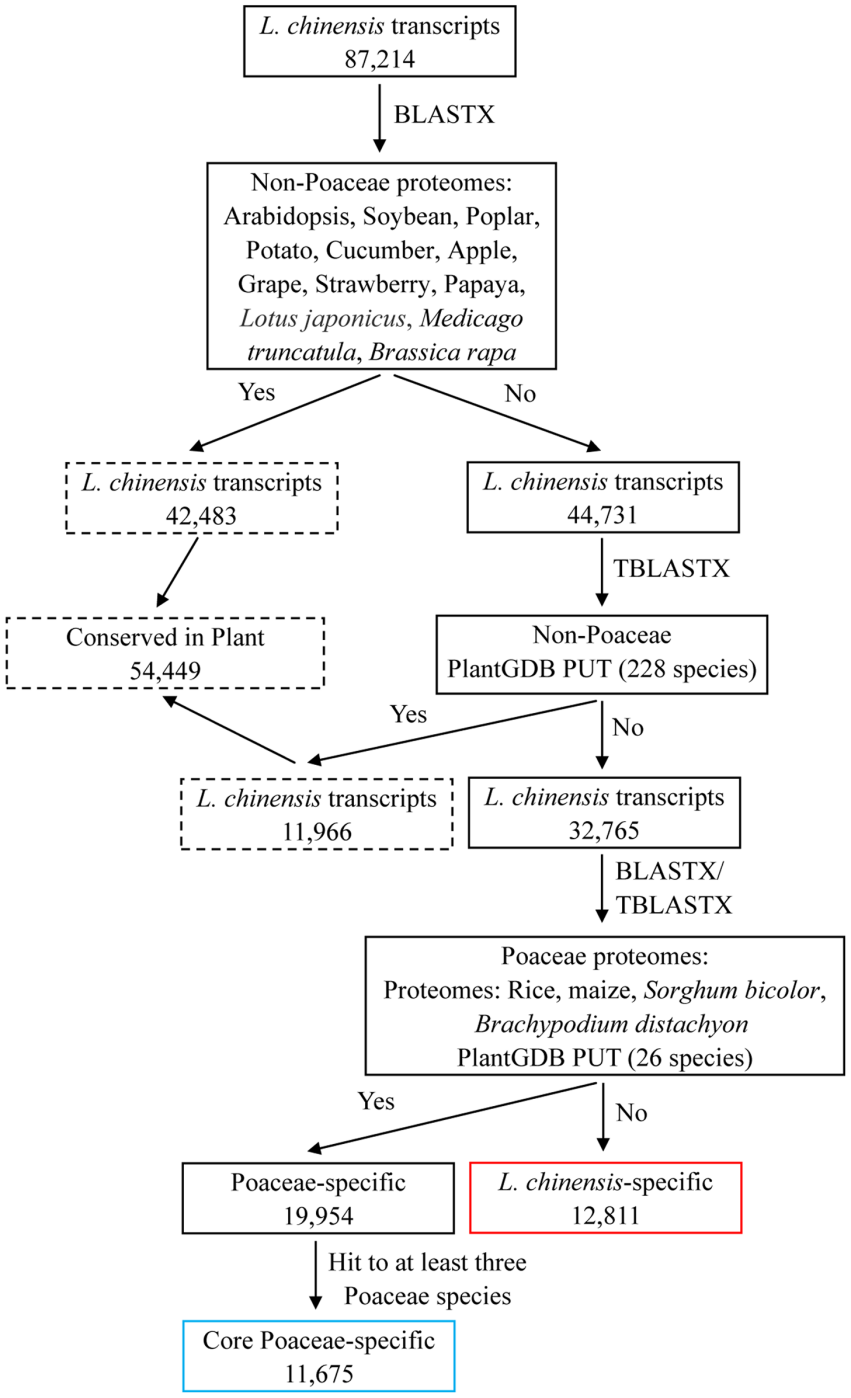
Strategy for the identification of Poaceae-specific genes and putatively novel sheepgrass genes. Transcripts that showed significant hits with non-Poaceae plant species are in dotted boxes. ‘Yes’ represents a significant hit and ‘No’ represents no significant hit in BLAST searches for the given criteria (E ≤1e-5 for BLASTX and TBLASTX). The sheepgrass unigenes identified as putatively novel sheepgrass genes and core Poaceae-specific genes are highlighted in red and blue boxes, respectively.

To identify Poaceae-specific unigenes and putatively novel sheepgrass unigenes, a series of BLAST searches were performed using an E threshold of 1e-5 and protein identity no less than 30%. Figure 8 is a summary of the analysis processes and results obtained. In the first step, 42,483 sheepgrass unigenes that showed BLASTX hits with the protein sequences of 12 annotated non-Poaceae plant genomes were removed. In the second step, the remaining 44,731 sheepgrass unigenes were searched via TBLASTX against non-Poaceae plant transcript assemblies from 228 species available at PlantGDB PUT (Table S2); 11,966 unigenes that showed a significant hit with at least one of these sequences were removed. Thus, a total of 54,449 unigenes were removed and considered to be conserved in the above analyzed non-Poaceae plant species. Subsequently, a BLASTX search was performed against the protein sequences of rice, *Brachypodium*, corn and sorghum, and a TBLASTX search was performed against transcript assemblies of 26 Poaceae species available at PlantGDB PUT (Table S2). A total of 19,954 unigenes showed significant similarity with at least one of the above sequences and were considered to be candidate Poaceae-specific unigenes. Among these, 11,675 unigenes were predicted to be core Poaceae-specific unigenes that showed significant similarity with at least 3 of the above sequences. Another 12,811 unigenes did not show a significant hit with any of the above sequences and represent putatively novel sheepgrass genes. To explore if these sequences without BLAST hits were from UTRs or non-coding RNAs, the transcript sequences were searched against the RFam database version 11 using Infernal [32], [33], with the hit inclusion cutoff set as the TC (trusted cutoff) bit score threshold in the Rfam model. This analysis showed a match of only 0.07%, which indicated that most of the sequences without BLAST hits were from coding sequences and represented sheepgrass putative novel genes.

### Freezing Stress-responsive Unigenes in Sheepgrass

**Figure 9:**
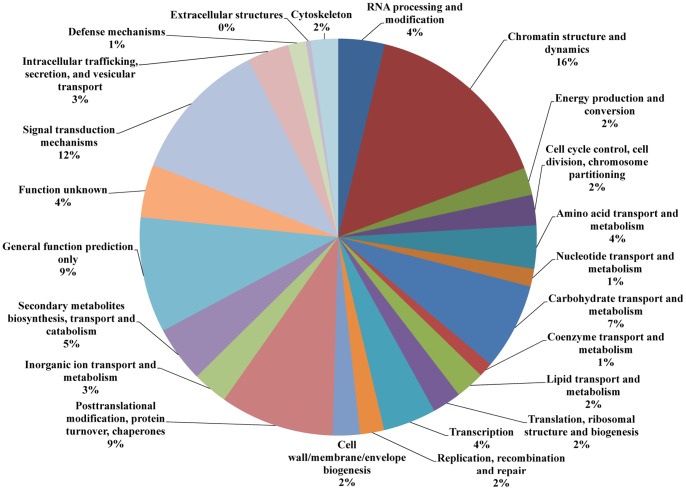
Classification of KOG function for sheepgrass freezing stress-responsive unigenes.

A total of 2,979 unigenes had significantly induced or inhibited expression when tissues were treated with freezing conditions (−40°C and −15°C) compared to control tissues (25°C) (Table S3), as assessed by the differentially expressed gene analysis described in the Materials and Methods. KOG functional classification indicated that a number of these unigenes were assigned to the categories “chromatin structure and dynamics” (16%), “signal transduction mechanisms” (12%), “posttranslational modification, protein turnover, chaperones” (9%), “general function prediction only” (9%), and “carbohydrate transport and metabolism” (7%) (Figure 9). In the category of “chromatin structure and dynamics”, the unigenes were histones, including Histone 2A, Histone H2B, Histone H3 and Histone H4. In the category of “signal transduction mechanisms”, most of unigenes encoded various protein kinases, Apoptotic ATPase, and the RhoA GTPase effector DIA/Diaphanous. Among identified 2,979 freezing-responsive unigenes in sheepgrass, many unigenes were known CBF-dependent and independent genes by comparison with the freezing-responsive genes reported in *Arabidopsis* and other Poaceae species, including CBF genes and their regulon, direct regulators of CBF/DREB1 expression (such as *ICE1*, *ICE2* and *MYB15*), and CBF-independent genes (such as *HOS10*, *FRY2*, *LOS2* and *ESK1*) (Table S4), but we also found 366 unigenes that were putatively novel sheepgrass freezing-responsive genes.

### Identification of Simple Sequence Repeats (SSRs)

To identify SSRs in sheepgrass, we used the perl script MISA program to mine the transcripts generated in this study. Dinucleotides that repeated more than six times and tri-, tetra-, penta- and hexanucleotides that repeated more than five times were considered as search criteria for SSRs. A total of 3,818 SSRs were identified in 87,214 sheepgrass unigenes. Overall, 3,597 unigenes were SSR-containing sequences, including 220 sequences with more than 1 SSR and 131 SSRs present in a compound formation (Table S5). On average, there was one SSR locus per 10.78 kb of sheepgrass transcriptome sequence (Table 3). Trinucleotide repeats made up the highest proportion of SSRs, with a frequency of 74.12%, followed by di- (22.29%) and tetranucleotide (2.67%) repeats. The lowest fraction of SSRs were penta- (0.37%) and hexanucleotide (0.55%) repeats (Table 3). Among the SSRs identified, motifs of CCG/CGG, AGC/CTG and AGG/CCT were represented in the trinucleotide repeats, and motifs of AG/CT and AC/GT were dominant in the dinucleotide repeats (Table 3). Among the SSR-containing unigenes, there were 109 unigenes that were also responsive to freezing; this finding indicated that they were candidate freezing stress-related EST-SSRs and worthy of further study.

**Table 3:** Summary of EST-SSRs identified in sheepgrass transcripts.

Searching items	Numbers/Percentage (%)
SSR mining	
Total number of sequences examined a	87,214
Total size of examined sequences (bp)	41,139,817
Total number of identified SSRs	3,818
Number of SSR containing sequences (%)	3,597/4.12
Number of sequences containing more than 1 SSR	220
Number of SSRs present in compound formation b	131
Frequency of SSRs	One per 10.78kb
SSRs/transcripts (%)	4.38
Distribution of SSRs in different repeat types	
Di-nucleotide	851/22.29
Tri-nucleotide	2,830/74.12
Tetra-nucleotide	102/2.67
Penta-nucleotide	14/0.37
Hexa-nucleotide	21/0.55
Frequencies of different motifs in di- and tri-nucleotide repeats	
AC/GT	262/30.79
AG/CT	414/48. 65
AT/AT	84/9.87
CG/CG	91/10.69
AAC/GTT	61/2.16
AAG/CTT	195/6.89
AAT/ATT	28/0.99
ACC/GGT	172/6.08
ACG/CGT	181/6.40
ACT/AGT	24/0.85
AGC/CTG	458/16.18
AGG/CCT	478/16.89
ATC/ATG	109/3.85
CCG/CGG	1,124/39.72

a All assembled contigs 100 bp and longer and singletons 300 bp and longer.

b Multiple SSRs in one transcript separated by fewer than 100 bp were defined as being in compound formation.

### Similarity and Phylogenetic Analysis of Sheepgrass with Other Poaceae Species

**Figure 10:**
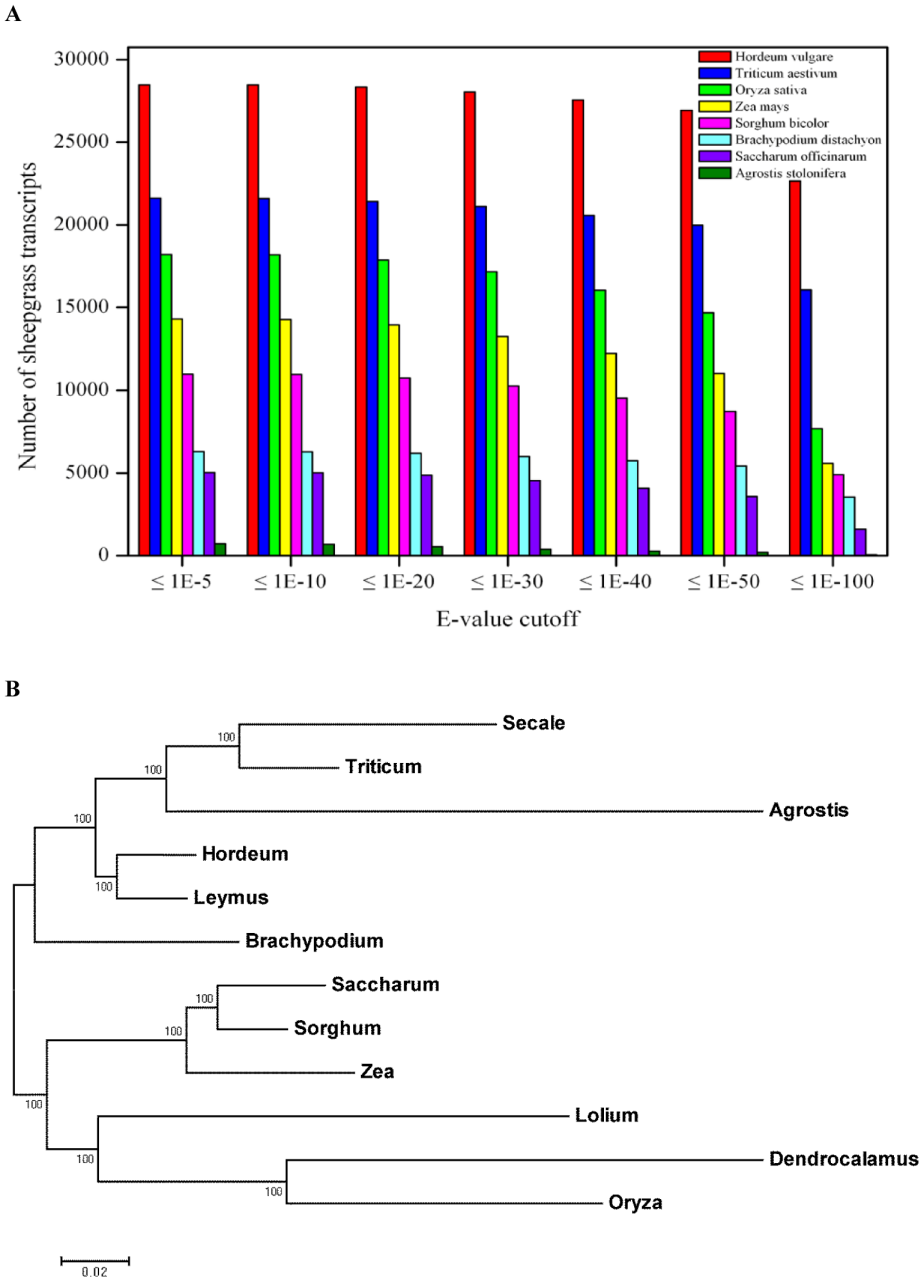
Similarity and phylogenetic analysis of sheepgrass with other Poaceae species. (A) Similarity analysis of sheepgrass with other Poaceae species. (B) Relationship of sheepgrass to other grasses. Rooted phylogenetic tree based on the combined partial nucleotide sequences of 29 highly expressed genes.

The extent of gene conservation was determined by using BLASTN to compare sheepgrass unigenes with the corresponding unigenes of other Poaceae species from NCBI, with a threshold E-value of 1e-5 to 1e-100. At any similarity level, sheepgrass unigenes showed a higher hit number with barley and wheat unigenes than that with other Poaceae species unigenes (Figure 10A). To better establish the relationship of sheepgrass to other grasses, we created a phylogenetic tree based on partial sequences from 29 highly expressed genes in sheepgrass (details see Methods). Then, we used these genes to find the corresponding genes in other Poaceae species included in the analysis (Table S6). The aligned sequences of all 29 genes were combined to produce one consensus sequence for each species that was used for phylogenetic analysis (Table S7). Using this alignment, phylogenetic trees were created using programs from MegAlign, ClustalX, and the PHYLIP software package. Our phylogenetic analysis from 29 genes indicated that sheepgrass is more closely related to barley and wheat than to *Brachypodium*, rice, corn, sugarcane or sorghum (Figure 10B).

## Discussion

As an important non-model Poaceae forage grass with a very large genome, sheepgrass has insufficient transcriptomic and genomic data in public databases. The development of genomic resources using non-model species will allow their gene discovery, the development of molecular markers, the determination of phylogenetic relationships, and the genetic analysis of adaptive traits. The Roche GS FLX NGS platform has proven to be valuable for non-model plant systems, such as olive [34], chestnut [35], *Artemisia annua* [36], ginseng [37], strawberry [38], bracken fern [39], switchgrass [40], [41], and *Phragmites australis* [42]. Here, we used the Roche GS FLX high-throughput sequencing technology to profile the sheepgrass transcriptome. Five cDNA libraries were constructed using RNA samples from mixed tissues of various developmental stages and stress treatments, buds from freezing stress treatments (−40°C, −15°C) and control treatments (25°C), and spikes from different developmental stages. This method helps to increase the number of sheepgrass-expressed transcripts included in the analysis, especially those related to freezing resistance. Although a saline-alkaline treatment transcriptome has been reported in sheepgrass using Roche-454 massive parallel pyrosequencing technology [31], the significant contributions in this study are that a lot of freezing-response related genes in sheepgrass were identified, as well as many EST-SSRs, including some SSRs related to sheepgrass freezing-responsive genes, were also identified for potential gene-phenotype study in future.

Accurate sequencing and a reliable read assembly are essential for downstream analysis and applications of transcriptome data. In this study, we used several de novo assembly programs, including CAP3, CLC Genomics workbench (version 3.7.1), MIRA (version 3.2.0), and Newbler (v2.3 and v2.5p1), to obtain the best assembly results (data not shown). According to these assembly results, we determined that the assembly of Newbler 2.5 (pl) was better than other programs when considering several criteria, including N50, contig mean length, reads used, uniquely mapped reads, and similarity/coverage to the reference sequences (*Brachypodium distachyon* and rice). This comparative analysis was similar with Kumar and Blaxter (2010) [43], who showed that Newbler 2.5 gave longer contigs, better alignments to some reference sequences and was fast and easy to use compared with other assemblers. A majority (approximately 88%) of reads was assembled into 32,416 contigs using the Newbler 2.5 (pl) assembler, and the assembled efficiencies were high and comparable to a similar studies (88% [44], 90% [45]). A large number of singletons (54,798) were also obtained. Most contigs and singletons were clean and from sheepgrass tissues by our analysis, indicating that template contamination is not the cause of singleton generation.

Freezing tolerance in plants is a critical factor that limits the geographic distribution of wild species [46]. Sheepgrass is a species with a strong freezing tolerance. In this study, the 2,979 identified unigenes with differential responsiveness to freezing were assigned to various categories. In the most represented category, “chromatin structure and dynamics”, the main unigenes were histones. In plants, histone modification (acetylation/deacetylation) has been shown to be involved in metastable (epigenetic) changes required to maintain altered cellular and tissue properties after several rounds of mitosis [47]. In fact, histone modification has been shown to control the cold-induced (vernalization) flowering response and to play a critical role in gene activation/repression in plant acclimation and tolerance to freezing [48], [49].

The cold signal in plants activates CBF-dependent and CBF-independent transcriptional pathways [50]. In *Arabidopsis thaliana*, three CBF genes, *CBF1*, *CBF2*, and *CBF3*, as well as genes induced by CBF (termed the CBF regulon), are induced when plants are exposed to low temperature [51]–[53]. Direct regulators of CBF/DREB1 expression are *HOS1*, *ICE1*, *ICE2* and *MYB15* [54]–[56]. *HOS10*, *FRY2*, *LOS2* and *ESK1* are CBF-independent transcriptional pathways in *Arabidopsis* [57]–[60]. In this study, we found many putative CBF-dependent and CBF-independent unigenes from sheepgrass by BLAST searches against reported genes in *Arabidopsis* and Poaceae species [61], [62] (Table S4). This analysis may indicate that plants have some conserved mechanism to induce common pathways such as CBF in response to low temperature stress.

In addition to conserved pathway genes, 366 unigenes were found to be putatively novel freezing responsive genes that lacked homologues in other lineages and were also called orphan genes [63]. Orphan genes might arise from duplication and rearrangement processes followed by fast divergence, and these genes are thought to be particularly important for taxon-specific developmental adaptations and interactions with the environment as a consequence of lineage-specific adaptations [64]–[66]. Our results indicate that sheepgrass harbors a large number of putative orphan genes (12,811 unigenes), and 366 unigenes represent a small part of the orphan genes, which become specifically activated in response to freezing stress and are worthy of further study to explore if they indicate a new mechanism of sheepgrass freezing tolerance.

Microsatellites, or simple sequence repeats (SSRs), are 1–6 bp iterations of DNA sequences that are known to occur only in non-coding regions. However, the occurrence of SSRs in transcribed sequences is now well established, and they are commonly known as EST-SSRs or genic SSRs. These SSRs have been reported in a number of Poaceae species such as rice [67], bread wheat [68], barley [69], sugarcane [70], and the hybrid progeny of *Leymus cinereus*×*Leymus triticiodes* [71]. However, no EST-SSRs have been reported in sheepgrass until now.

In this study, we identified 3,818 EST-SSRs from 87,214 unigenes in sheepgrass. The frequency of SSR per sheepgrass unigene was 4.38% (Table 3), which was slightly higher than rice (3.57%) and much higher than *Arabidopsis* (0.84%) [72]. We found that trinucleotide sequences represented the highest proportion of SSRs, with a frequency of 74.12%. Our results supported an earlier report showing that trimer motifs of EST-SSRs were more frequent in the majority of higher plant groups, such as monocots and dicots, whereas dimer motifs were more frequent in lower plant species, such as green algae and mosses [72]. The enhanced frequency of trinucleotide repeats in the coding sequences of many organisms is a sign of the effects of selection, indicating that those SSRs were selected against possible frameshift mutations. Among the SSRs identified in this study, motifs of CCG were predominant for the trinucleotide repeats, followed by AGG and AGC, similar to in rice, barley and wheat, as previous described [73], [74]. For dinucleotides, our results were the same as those for wheat and barley, as AG and AC were the most common sequences in wheat and barley EST-SSRs. In this study, the identified 109 freezing responsive and EST-SSR containing unigenes mainly encoded regulatory proteins, such as transcription factors and protein kinases; this finding indicates that SSRs are a factor contributing to the fast evolution of adaptive phenotypes, as reported by Young et al. (2000) [75]. In plants and other species, outcomes of SSR variation within their genes remain to be further studied. Future studies might address the significant evolutionary role of SSRs in regulating gene expression under diverse environmental stresses [76].

*Leymus* species have two basic genomes: Ns and Xm [77]. Previous studies based on morphology, cytogenetics, DNA hybridization patterns, and DNA sequences (nrITS, trnL-F) have revealed that the Ns genome of *Leymus* originated from the genus *Psathyrostachys* [1], [77]–[79]. The origin of the Xm genome in *Leymus* is controversial. It may have originated from *Elymus californicus* or an ancestral lineage of *Agropyron* and *Eremopyrum triticeum* [1], [80]. The relationship of sheeepgrass in *Leymus* to other important Poaceae species, especially crops, is poorly understood. In this study, we considered that sheepgrass is more closely related to barley and wheat than to *Brachypodium*, rice, corn, sugarcane or sorghum based on similarity and phylogenetic analysis (Figure 10). It has been suggested that *Brachypodium* and rice diverged 40 million years ago. *Brachypodium* and its closest *Triticeae* relative diverged 25 to 30 million years ago [81], [82]. Wheat and barley diverged 11.6 million years ago based on sequences of *Acc* and other genes [83]. Molecular dating based on intron data of the *Acc1* gene also dated the most recent common ancestor (MRCA) of *Leymus* to 11–12 million years ago, indicating that the time of barley and wheat divergence might be very close to sheepgrass.

### Conclusions

As an important perennial forage grass across the Eurasian Steppe, sheepgrass is known for its adaptability to various environmental conditions. Insufficient transcriptomic and genomic data in public databases have limited our understanding of the molecular mechanism underlying the multiple-stress tolerance of sheepgrass. The 87,214 unigenes in this 454 EST collection have enriched the sheepgrass transcriptomic-level resources and will be useful for further comparative and functional genomic studies in the *Leymus* genus and Poaceae species. The potentially novel sheepgrass genes and freezing stress-responsive genes identified in this study provide a foundation for further investigation into the genetic basis of the environmental and ecological adaptations of this species. The stress resistance genes will also be used to improve wheat and barley crops through hybridization or genetic transformation, as wheat and barley have very close relationships to sheepgrass. The thousands of EST-SSRs identified here will be a valuable resource for future gene-phenotype studies as well as for the molecular breeding of sheepgrass and other Poaceae species.

## Materials and Methods

### Plant Materials

All sheepgrass materials (variety Zhongke No. 2) were obtained from field- or growth chamber-grown plants. Tissues for different developmental stages of leaves, sheaths, rhizomes, and roots as well as spikes from booting to maturity were collected in the field. For stress treatments, sheepgrass was grown in soil mix of peat moss and vermiculite (2∶1, v/v) in the greenhouse at an average temperature of 25°C under long-day conditions (16 h light/8 h dark). 8-week-old seedlings of sheepgrass were irrigated with 400 mM NaCl for salinity stress, and cut to 25%, 50% and 75%, respectively, for defoliation treatments. The treated shoots and roots were harvested at 4, 8, 12, 24 and 48 h. Since temperatures of −40°C and −15°C were the approximate extremely low temperatures of sheepgrass grown in its main distribution areas and in Beijing experimental field, 3-month-old plants with a large number of tiller and root buds were treated with cold acclimation at 4°C for 3 days and then frozen at −40°C and −15°C in the dark, respectively, and plants grown at 25°C in the dark was used as a control. The freezing treated tiller and root buds were collected at 4, 8, 12, 24 and 48 h. At least three independent biological replicates of each tissue sample were harvested and immediately frozen in liquid nitrogen and stored at −80°C.

### RNA Isolation, Library Preparation and 454 Sequencing

Total RNA from each frozen sheepgrass tissue was extracted using the TRIzol Reagent according to the manufacturer’s instructions (Invitrogen, Carlsbad, CA, USA). The quality of total RNAs was checked using a NanoDrop 2000 (Thermo Fisher, USA). The mRNA was purified from total RNA samples using the Dynabead mRNA purification kit according to the manufacturer’s instructions (Invitrogen, Carlsbad, CA, USA), and the quality was assessed using the Aligent 2100 Bioanalyzer (Agilent Technologies, Inc., Waldbronn, Germany). Double-stranded cDNA was synthesized using the SuperScript Double-Stranded cDNA Synthesis kit (Invitrogen, Carlbad, CA, USA). Specific adapters were ligated to the fragmented cDNA and denatured to generate single-stranded cDNA, followed by emulsion PCR amplification for sequencing. Five cDNA libraries were generated. One cDNA library was made from mRNA isolated from different developmental stages of leaves, sheaths, rhizomes, and roots as well as from conditions of defoliation and salinity stress. Three cDNA libraries were made from mRNA separately isolated from tiller buds and root buds at different temperature conditions (−40°C, −15°C and 25°C). The fifth cDNA library was made from mRNA pooled in equal amounts from spikes at different developmental stages, from booting to maturity. Library quality was assessed using the High Sensitivity DNA kit on an Agilent 2100 Bioanalyzer. All five cDNA libraries were sequenced using the Roche GS FLX Titanium sequencing reagents and sequencer.

### Sequence Filtering and *de novo* Assembly

Roche/454 sequence reads generated in this study were deposited in the NCBI sequence read archive (SRA065691). Sequences were prepared for assembly by Q20 filtering, removal of library adapter sequences using estclean (https://sourceforge.net/projects/estclean/) and a custom perl script, removal of contaminating vector and poly A/T stretches using SeqClean (http://compbio.dfci.harvard.edu/tgi/software/), and removal of short reads (<60 bp) and reads belonging to mitochondria and plastids by comparison with plant mitochondria and plastid genome sequences from NCBI (www.ncbi.nlm.nih.gov/). All high quality reads were assembled using the *de novo* assembler Newbler version 2.5(p1) (http://www.454.com/products-solutions/analysistools/gs-de-novo-assembler.asp) with the cDNA option using multiple CPUs.

### Annotation, Functional Classification and Pathway Analysis

All assembled unigenes were annotated with GetORF from the EMBOSS package [84]. The ORF of each predicted protein was used for BLASTP searches against the Swiss-Prot and NCBI nr databases with thresholds of E-value ≤1e-5. Domain-based alignments were carried out against the KOG database at NCBI with a cut-off E-value of ≤1e-5. GO annotations for describing biological process, molecular functions, and cellular components were analyzed by GoPipe using a BLASTP search against the Swiss-Prot and TrEMBL databases with an E-value ≤1e-5 [85]. KEGG pathways annotations were performed using the KEGG Automatic Annotation Server (KAAS) with the bi-directional best-hit information method [86]. KAAS annotates every submitted sequence with KEGG orthology (KO) identifiers that represent an orthologous group of genes directly linked to an object in the KEGG pathways and BRITE functional hierarchy [86], [87]. Transcription factors (TFs) were analyzed with all unigenes by BLASTX searches against the Plant Transcription Factor Database (version 3.0) (E-value ≤1e-10).

### Identification of Poaceae-specific and Species-specific Genes

The plant transcript assemblies for non-Poaceae/Poaceae plant species were downloaded from the PlantGDB EST Assemblies database (http://www.plantgdb.org/). The dataset of transcript assemblies was comprised of 228 non-Poaceae and 26 Poaceae species. The proteomes sequences for all of the completely sequenced plants were downloaded from their respective genome project websites (for these plant species, see Figure 6). Criteria of an E-value ≤1e-5 for BLASTX and TBLASTX searches were used for filtering significant hits. In-house perl scripts were used for filtering the BLAST results of significant and non-significant hits and their sequences.

### Analysis of Differentially Expressed Genes (DEGs)

To analyze freezing–responsive, differentially expressed genes in sheepgrass, the number of reads for each of the contigs from the three samples of freezing treatments (−40°C, −15°C and 25°C) was converted to reads per kilobase per million (RPKM) [88]. Then, the MA-plot-based method with Random Sampling model (MARS) in the DEGseq package was used to calculate the expression abundance of each contig between the analyzed samples. We used an FDR (false discovery rate) to determine the p value threshold. An FDR<0.001 was considered to have a significant expression abundance.

### EST-SSR Identification

The MIcroSAtellite (MISA; http://pgrc.ipk-gatersleben.de/misa/) program was used to identify SSRs. Dinucleotides that were repeated more than six times or tri-, tetra-, penta- or hexanucleotide that were repeated more than five times were considered as search criteria for SSRs in MISA script.

### Similarity and Phylogenetic Analysis

The extent of gene conservation was determined by BLASTN analysis comparing unigenes of barley, wheat, *Brachypodium distachyon*, rice, corn, sorghum, *Saccharum officinarum* and *Agrostis stolonifera* downloaded from NCBI to sheepgrass unigenes at a similarity level of ≤1E-5 to ≤1E-100, an identity of 80%, and a coverage of 50%.

We selected 29 highly expressed, suitable genes from sheepgrass transcripts for phylogenetic analysis. First, candidate contigs with highly significant hits to known genes were selected by comparison to the NCBI non-redundant database using the BLASTN algorithm. Then, the corresponding genes were retrieved from barley (Hordeum vulgare), wheat (Triticum aestivum), *Brachypodium* (*Brachypodium distachyon*), rice (*Oryza sativa*), sorghum (*Sorghum bicolor*), corn (*Zea mays*), *Agrostis stolonifera*, *Dendrocalamus latiflorus*, *Lolium perenne*, *Saccharum officinarum* and *Secale cereal* from the EST or protein database using BLASTN or BLASTX. The coding sequences from the 29 highly expressed genes selected above were aligned. The aligned sequences were then combined to produce one sequence for each species that was used for phylogenetic analysis. Phylogenetic trees were constructed based on this alignment using MegAlign, ClustalX version 1.81, and the PHYLIP software package version 3.69 (http://evolution.genetics.washington.edu/phylip.html) [89].

## Supporting Information

Table S1 **KEGG pathways identified in sheepgrass transcripts.**
(DOC)

Table S2 **A list of 228 non-Poaceae plant species and 26 Poaceae species available at PlantGDB PUT.**
(XLS)

Table S3 **A list of 2,979 DEGs under freezing stress and their annotation characterization.**
(XLS)

Table S4 **Putative CBF-dependent and CBF-independent unigenes identified from sheepgrass transcriptome and the corresponding ortholog genes in** ***Arabidopsis*** **and Poaceae species.**
(DOC)

Table S5 **A list of EST-SSRs contained unigenes and characterizations.**
(XLS)

Table S6 **All 29 genes and their IDs in sheepgrass and other Poaceae species used for construction of phylogenetic tree.**
(XLS)

Table S7 **The aligned and combined sequences of all 29 genes for sheepgrass and other Poaceae species.**
(FAS)
