# Supplementary material for: Transcriptome Analysis in Sheepgrass (Leymus chinensis): A Dominant Perennial Grass of the Eurasian Steppe
Source: PLoS One. 2013 Jul 4;8(7):e67974. doi: 10.1371/journal.pone.0067974 (PMC3701641; doi:10.1371/journal.pone.0067974)
Supplement: Table S1 — KEGG pathways identified in sheepgrass transcripts. (DOC) [file pone.0067974.s001.doc]

Pathway KO Number *L. chinensis* unigenes

1. Metabolism

Carbohydrate Metabolism 270 579

[Glycolysis / Gluconeogenesis](http://www.genome.jp/kegg/pathway/map/map00010.html) 30 65

[Citrate cycle (TCA cycle)](http://www.genome.jp/kegg/pathway/map/map00020.html) 20 36

[Pentose phosphate pathway](http://www.genome.jp/kegg/pathway/map/map00030.html) 14 41

[Pentose and glucuronate interconversions](http://www.genome.jp/kegg/pathway/map/map00040.html) 11 32

[Fructose and mannose metabolism](http://www.genome.jp/kegg/pathway/map/map00051.html) 16 30

[Galactose metabolism](http://www.genome.jp/kegg/pathway/map/map00052.html) 14 27

[Ascorbate and aldarate metabolism](http://www.genome.jp/kegg/pathway/map/map00053.html) 13 35

[Starch and sucrose metabolism](http://www.genome.jp/kegg/pathway/map/map00500.html) 29 81

[Amino sugar and nucleotide sugar metabolism](http://www.genome.jp/kegg/pathway/map/map00520.html) 35 67

[Pyruvate metabolism](http://www.genome.jp/kegg/pathway/map/map00620.html) 23 53

[Glyoxylate and dicarboxylate metabolism](http://www.genome.jp/kegg/pathway/map/map00630.html) 14 29

[Propanoate metabolism](http://www.genome.jp/kegg/pathway/map/map00640.html) 15 28

[Butanoate metabolism](http://www.genome.jp/kegg/pathway/map/map00650.html) 14 24

[C5-Branched dibasic acid metabolism](http://www.genome.jp/kegg/pathway/map/map00660.html) 4 4

[Inositol phosphate metabolism](http://www.genome.jp/kegg/pathway/map/map00562.html) 18 27

Energy Metabolism 176 304

[Oxidative phosphorylation](http://www.genome.jp/kegg/pathway/map/map00190.html) 67 105

[Photosynthesis](http://www.genome.jp/kegg/pathway/map/map00195.html) 26 39

[Photosynthesis - antenna proteins](http://www.genome.jp/kegg/pathway/map/map00196.html) 11 14

[Carbon fixation in photosynthetic organisms](http://www.genome.jp/kegg/pathway/map/map00710.html) 21 50

[Methane metabolism](http://www.genome.jp/kegg/pathway/map/map00680.html) 23 52

[Nitrogen metabolism](http://www.genome.jp/kegg/pathway/map/map00910.html) 18 29

[Sulfur metabolism](http://www.genome.jp/kegg/pathway/map/map00920.html) 10 15

Lipid Metabolism 160 269

[Fatty acid biosynthesis](http://www.genome.jp/kegg/pathway/map/map00061.html) 9 11

[Fatty acid elongation](http://www.genome.jp/kegg/pathway/map/map00062.html) 7 16

[Fatty acid metabolism](http://www.genome.jp/kegg/pathway/map/map00071.html) 12 31

[Synthesis and degradation of ketone bodies](http://www.genome.jp/kegg/pathway/map/map00072.html) 4 9

[Cutin, suberine and wax biosynthesis](http://www.genome.jp/kegg/pathway/map/map00073.html) 4 6

[Steroid biosynthesis](http://www.genome.jp/kegg/pathway/map/map00100.html) 17 25

[Steroid hormone biosynthesis](http://www.genome.jp/kegg/pathway/map/map00140.html) 2 12

[Glycerolipid metabolism](http://www.genome.jp/kegg/pathway/map/map00561.html) 20 26

[Glycerophospholipid metabolism](http://www.genome.jp/kegg/pathway/map/map00564.html) 32 52

[Ether lipid metabolism](http://www.genome.jp/kegg/pathway/map/map00565.html) 7 15

[Sphingolipid metabolism](http://www.genome.jp/kegg/pathway/map/map00600.html) 11 14

[Arachidonic acid metabolism](http://www.genome.jp/kegg/pathway/map/map00590.html) 6 8

[Linoleic acid metabolism](http://www.genome.jp/kegg/pathway/map/map00591.html) 5 6

[alpha-Linolenic acid metabolism](http://www.genome.jp/kegg/pathway/map/map00592.html) 10 16

[Biosynthesis of unsaturated fatty acids](http://www.genome.jp/kegg/pathway/map/map01040.html) 14 22

Nucleotide Metabolism 147 217

[Purine metabolism](http://www.genome.jp/kegg/pathway/map/map00230.html) 82 125

[Pyrimidine metabolism](http://www.genome.jp/kegg/pathway/map/map00240.html) 65 92

Amino Acid Metabolism 249 418

[Alanine, aspartate and glutamate metabolism](http://www.genome.jp/kegg/pathway/map/map00250.html) 24 38

[Glycine, serine and threonine metabolism](http://www.genome.jp/kegg/pathway/map/map00260.html) 24 37

[Cysteine and methionine metabolism](http://www.genome.jp/kegg/pathway/map/map00270.html) 26 53

[Valine, leucine and isoleucine degradation](http://www.genome.jp/kegg/pathway/map/map00280.html) 22 37

[Valine, leucine and isoleucine biosynthesis](http://www.genome.jp/kegg/pathway/map/map00290.html) 13 21

[Lysine biosynthesis](http://www.genome.jp/kegg/pathway/map/map00300.html) 13 19

[Lysine degradation](http://www.genome.jp/kegg/pathway/map/map00310.html) 11 23

[Arginine and proline metabolism](http://www.genome.jp/kegg/pathway/map/map00330.html) 39 62

[Histidine metabolism](http://www.genome.jp/kegg/pathway/map/map00340.html) 13 26

[Tyrosine metabolism](http://www.genome.jp/kegg/pathway/map/map00350.html) 12 19

[Phenylalanine metabolism](http://www.genome.jp/kegg/pathway/map/map00360.html) 14 30

[Tryptophan metabolism](http://www.genome.jp/kegg/pathway/map/map00380.html) 16 27

[Phenylalanine, tyrosine and tryptophan biosynthesis](http://www.genome.jp/kegg/pathway/map/map00400.html) 22 26

Metabolism of Other Amino Acids 60 107

[beta-Alanine metabolism](http://www.genome.jp/kegg/pathway/map/map00410.html) 16 26

[Taurine and hypotaurine metabolism](http://www.genome.jp/kegg/pathway/map/map00430.html) 3 3

[Phosphonate and phosphinate metabolism](http://www.genome.jp/kegg/pathway/map/map00440.html) 4 8

[Selenocompound metabolism](http://www.genome.jp/kegg/pathway/map/map00450.html) 9 14

[Cyanoamino acid metabolism](http://www.genome.jp/kegg/pathway/map/map00460.html) 7 9

[D-Glutamine and D-glutamate metabolism](http://www.genome.jp/kegg/pathway/map/map00471.html) 2 2

[D-Alanine metabolism](http://www.genome.jp/kegg/pathway/map/map00473.html) 1 1

[Glutathione metabolism](http://www.genome.jp/kegg/pathway/map/map00480.html) 18 44

Glycan Biosynthesis and Metabolism 101 152

[N-Glycan biosynthesis](http://www.genome.jp/kegg/pathway/map/map00510.html) 31 44

[Various types of N-glycan biosynthesis](http://www.genome.jp/kegg/pathway/map/map00513.html) 20 30

[Other types of O-glycan biosynthesis](http://www.genome.jp/kegg/pathway/map/map00514.html) 3 13

[Glycosaminoglycan biosynthesis - chondroitin sulfate](http://www.genome.jp/kegg/pathway/map/map00532.html) 1 1

[Glycosaminoglycan biosynthesis - heparan sulfate](http://www.genome.jp/kegg/pathway/map/map00534.html) 2 2

[Glycosaminoglycan degradation](http://www.genome.jp/kegg/pathway/map/map00531.html) 5 8

[Glycosylphosphatidylinositol(GPI)-anchor biosynthesis](http://www.genome.jp/kegg/pathway/map/map00563.html) 20 26

[Glycosphingolipid biosynthesis - lacto and neolacto series](http://www.genome.jp/kegg/pathway/map/map00601.html) 1 1

[Glycosphingolipid biosynthesis - globo series](http://www.genome.jp/kegg/pathway/map/map00603.html) 2 3

[Glycosphingolipid biosynthesis - ganglio series](http://www.genome.jp/kegg/pathway/map/map00604.html) 2 3

[Lipopolysaccharide biosynthesis](http://www.genome.jp/kegg/pathway/map/map00540.html) 6 8

[Peptidoglycan biosynthesis](http://www.genome.jp/kegg/pathway/map/map00550.html) 1 1

[Other glycan degradation](http://www.genome.jp/kegg/pathway/map/map00511.html) 7 12

Metabolism of Cofactors and Vitamins 137 210

[Thiamine metabolism](http://www.genome.jp/kegg/pathway/map/map00730.html) 8 9

[Riboflavin metabolism](http://www.genome.jp/kegg/pathway/map/map00740.html) 7 8

[Vitamin B6 metabolism](http://www.genome.jp/kegg/pathway/map/map00750.html) 6 6

[Nicotinate and nicotinamide metabolism](http://www.genome.jp/kegg/pathway/map/map00760.html) 11 18

[Pantothenate and CoA biosynthesis](http://www.genome.jp/kegg/pathway/map/map00770.html) 16 27

[Biotin metabolism](http://www.genome.jp/kegg/pathway/map/map00780.html) 4 5

[Lipoic acid metabolism](http://www.genome.jp/kegg/pathway/map/map00785.html) 2 4

[Folate biosynthesis](http://www.genome.jp/kegg/pathway/map/map00790.html) 14 22

[One carbon pool by folate](http://www.genome.jp/kegg/pathway/map/map00670.html) 11 14

[Retinol metabolism](http://www.genome.jp/kegg/pathway/map/map00830.html) 7 18

[Porphyrin and chlorophyll metabolism](http://www.genome.jp/kegg/pathway/map/map00860.html) 32 52

[Ubiquinone and other terpenoid-quinone biosynthesis](http://www.genome.jp/kegg/pathway/map/map00130.html) 19 27

Metabolism of Terpenoids and Polyketides 58 91

[Terpenoid backbone biosynthesis](http://www.genome.jp/kegg/pathway/map/map00900.html) 21 33

[Monoterpenoid biosynthesis](http://www.genome.jp/kegg/pathway/map/map00902.html) 2 2

[Diterpenoid biosynthesis](http://www.genome.jp/kegg/pathway/map/map00904.html) 2 3

[Carotenoid biosynthesis](http://www.genome.jp/kegg/pathway/map/map00906.html) 14 18

[Brassinosteroid biosynthesis](http://www.genome.jp/kegg/pathway/map/map00905.html) 2 2

[Insect hormone biosynthesis](http://www.genome.jp/kegg/pathway/map/map00981.html) 1 1

[Zeatin biosynthesis](http://www.genome.jp/kegg/pathway/map/map00908.html) 3 7

[Limonene and pinene degradation](http://www.genome.jp/kegg/pathway/map/map00903.html) 5 11

[Geraniol degradation](http://www.genome.jp/kegg/pathway/map/map00281.html) 3 4

[Biosynthesis of ansamycins](http://www.genome.jp/kegg/pathway/map/map01051.html) 1 4

[Polyketide sugar unit biosynthesis](http://www.genome.jp/kegg/pathway/map/map00523.html) 2 3

[Biosynthesis of siderophore group nonribosomal peptides](http://www.genome.jp/kegg/pathway/map/map01053.html) 1 1

[Biosynthesis of vancomycin group antibiotics](http://www.genome.jp/kegg/pathway/map/map01055.html) 1 2

Biosynthesis of Other Secondary Metabolites 64 101

[Phenylpropanoid biosynthesis](http://www.genome.jp/kegg/pathway/map/map00940.html) 15 35

[Stilbenoid, diarylheptanoid and gingerol biosynthesis](http://www.genome.jp/kegg/pathway/map/map00945.html) 5 9

[Flavonoid biosynthesis](http://www.genome.jp/kegg/pathway/map/map00941.html) 12 13

[Flavone and flavonol biosynthesis](http://www.genome.jp/kegg/pathway/map/map00944.html) 4 5

[Indole alkaloid biosynthesis](http://www.genome.jp/kegg/pathway/map/map00901.html) 1 2

[Isoquinoline alkaloid biosynthesis](http://www.genome.jp/kegg/pathway/map/map00950.html) 4 6

[Tropane, piperidine and pyridine alkaloid biosynthesis](http://www.genome.jp/kegg/pathway/map/map00960.html) 7 9

[Caffeine metabolism](http://www.genome.jp/kegg/pathway/map/map00232.html) 3 3

[Glucosinolate biosynthesis](http://www.genome.jp/kegg/pathway/map/map00966.html) 2 2

[Penicillin and cephalosporin biosynthesis](http://www.genome.jp/kegg/pathway/map/map00311.html) 1 1

[beta-Lactam resistance](http://www.genome.jp/kegg/pathway/map/map00312.html) 1 1

[Streptomycin biosynthesis](http://www.genome.jp/kegg/pathway/map/map00521.html) 5 9

[Butirosin and neomycin biosynthesis](http://www.genome.jp/kegg/pathway/map/map00524.html) 1 2

[Novobiocin biosynthesis](http://www.genome.jp/kegg/pathway/map/map00401.html) 3 4

Xenobiotics Biodegradation and Metabolism 50 101

[Benzoate degradation](http://www.genome.jp/kegg/pathway/map/map00362.html) 4 11

[Aminobenzoate degradation](http://www.genome.jp/kegg/pathway/map/map00627.html) 9 14

[Fluorobenzoate degradation](http://www.genome.jp/kegg/pathway/map/map00364.html) 1 2

[Chloroalkane and chloroalkene degradation](http://www.genome.jp/kegg/pathway/map/map00625.html) 5 8

[Chlorocyclohexane and chlorobenzene degradation](http://www.genome.jp/kegg/pathway/map/map00361.html) 2 3

[Toluene degradation](http://www.genome.jp/kegg/pathway/map/map00623.html) 1 2

[Nitrotoluene degradation](http://www.genome.jp/kegg/pathway/map/map00633.html) 1 1

[Styrene degradation](http://www.genome.jp/kegg/pathway/map/map00643.html) 3 3

[Atrazine degradation](http://www.genome.jp/kegg/pathway/map/map00791.html) 3 4

[Caprolactam degradation](http://www.genome.jp/kegg/pathway/map/map00930.html) 3 4

[DDT degradation](http://www.genome.jp/kegg/pathway/map/map00351.html) 1 1

[Bisphenol degradation](http://www.genome.jp/kegg/pathway/map/map00363.html) 3 6

[Naphthalene degradation](http://www.genome.jp/kegg/pathway/map/map00626.html) 3 4

[Polycyclic aromatic hydrocarbon degradation](http://www.genome.jp/kegg/pathway/map/map00624.html) 4 11

[Metabolism of xenobiotics by cytochrome P450](http://www.genome.jp/kegg/pathway/map/map00980.html) 7 27

#### Genetic Information Processing

Transcription 142 190

[RNA polymerase](http://www.genome.jp/kegg/pathway/ko/ko03020.html) 23 30

[Basal transcription factors](http://www.genome.jp/kegg/pathway/ko/ko03022.html) 27 32

[Spliceosome](http://www.genome.jp/kegg/pathway/ko/ko03040.html) 92 128

Translation 326 574

[Ribosome](http://www.genome.jp/kegg/pathway/ko/ko03010.html) 114 267

[Aminoacyl-tRNA biosynthesis](http://www.genome.jp/kegg/pathway/map/map00970.html) 26 52

[RNA transport](http://www.genome.jp/kegg/pathway/ko/ko03013.html) 85 117

[mRNA surveillance pathway](http://www.genome.jp/kegg/pathway/ko/ko03015.html) 43 67

[Ribosome biogenesis in eukaryotes](http://www.genome.jp/kegg/pathway/ko/ko03008.html) 58 71

Folding, Sorting and Degradation 249 384

[Protein export](http://www.genome.jp/kegg/pathway/ko/ko03060.html) 26 45

[Protein processing in endoplasmic reticulum](http://www.genome.jp/kegg/pathway/ko/ko04141.html) 64 106

[SNARE interactions in vesicular transport](http://www.genome.jp/kegg/pathway/ko/ko04130.html) 19 30

[Ubiquitin mediated proteolysis](http://www.genome.jp/kegg/pathway/ko/ko04120.html) 54 84

[Sulfur relay system](http://www.genome.jp/kegg/pathway/ko/ko04122.html) 9 9

[Proteasome](http://www.genome.jp/kegg/pathway/ko/ko03050.html) 33 49

[RNA degradation](http://www.genome.jp/kegg/pathway/ko/ko03018.html) 44 61

Replication and Repair 166 257

[DNA replication](http://www.genome.jp/kegg/pathway/ko/ko03030.html) 30 42

[Base excision repair](http://www.genome.jp/kegg/pathway/ko/ko03410.html) 25 42

[Nucleotide excision repair](http://www.genome.jp/kegg/pathway/ko/ko03420.html) 35 54

[Mismatch repair](http://www.genome.jp/kegg/pathway/ko/ko03430.html) 20 32

[Homologous recombination](http://www.genome.jp/kegg/pathway/ko/ko03440.html) 23 39

[Non-homologous end-joining](http://www.genome.jp/kegg/pathway/ko/ko03450.html) 8 9

[Fanconi anemia pathway](http://www.genome.jp/kegg/pathway/ko/ko03460.html) 25 39

#### Environmental Information Processing

Membrane Transport 8 12

[ABC transporters](http://www.genome.jp/kegg/pathway/ko/ko02010.html) 8 12

Signal Transduction 69 128

[Two-component system](http://www.genome.jp/kegg/pathway/ko/ko02020.html) 14 23

[MAPK signaling pathway](http://www.genome.jp/kegg/pathway/hsa/hsa04010.html) 11 23

[Calcium signaling pathway](http://www.genome.jp/kegg/pathway/hsa/hsa04020.html) 13 26

[Plant hormone signal transduction](http://www.genome.jp/kegg/pathway/ath/ath04075.html) 31 56

#### Cellular Processes

Transport and Catabolism 133 203

[Endocytosis](http://www.genome.jp/kegg/pathway/ko/ko04144.html) 30 46

[Phagosome](http://www.genome.jp/kegg/pathway/ko/ko04145.html) 28 46

[Lysosome](http://www.genome.jp/kegg/pathway/ko/ko04142.html) 34 49

[Peroxisome](http://www.genome.jp/kegg/pathway/ko/ko04146.html) 32 48

[Regulation of autophagy](http://www.genome.jp/kegg/pathway/ko/ko04140.html) *9* 14

Cell Growth and Death 79 111

[Cell cycle](http://www.genome.jp/kegg/pathway/hsa/hsa04110.html) 50 62

[Oocyte meiosis](http://www.genome.jp/kegg/pathway/xla/xla04114.html) 29 49

#### Organismal Systems

Sensory System 2 5

[Phototransduction](http://www.genome.jp/kegg/pathway/hsa/hsa04744.html) 2 5

Environmental Adaptation 23 51

[Plant-pathogen interaction](http://www.genome.jp/kegg/pathway/ath/ath04626.html) 23 51
