# Supplementary material for: Transcriptome Analysis in Sheepgrass (Leymus chinensis): A Dominant Perennial Grass of the Eurasian Steppe
Source: PLoS One. 2013 Jul 4;8(7):e67974. doi: 10.1371/journal.pone.0067974 (PMC3701641; doi:10.1371/journal.pone.0067974)
Supplement: Table S4 — Putative CBF-dependent and CBF-independent unigenes identified from sheepgrass transcriptome and the corresponding ortholog genes in Arabidopsis and Poaceae species. (DOC) [file pone.0067974.s004.doc]

| ***L.chinensis* unigenes (c:contig)** | **Genes in other plants** | **Locus** | **Species** | **Reference** |
| --- | --- | --- | --- | --- |
| c31221;c35607 | ICE1(inducer of CBF expression 1) | At3g26744 | *Arabidopsis* | [54] |
| c33672;c35031 | ICE2 | At1g12860 | *Arabidopsis* | [55] |
| c33806 | MYB15 | [At3g23250](http://arabidopsis.org/servlets/TairObject?type=locus&id=37690) | *Arabidopsis* | [56] |
| c07872 | HOS10(an R2R3-type MYB) | At1g35515 | *Arabidopsis* | [57] |
| c34036 | LOS2(a bifunctional enolase) | At2g36530 | *Arabidopsis* | [59] |
| c15843;c25330 | ESK1 | At3g55990 | *Arabidopsis* | [60] |
| c05149 | FRY2 | At4g21670 | *Arabidopsis* | [58] |
| c09990;c09993;c31655 | CBF3 | At4g25480 | *Arabidopsis* | [51] |
| c28499;c30030 | CBF1 | At4g25490 | *Arabidopsis* | [51] |
| c29132 | UTP-glucose glucosyltransferases | At1g01420 | *Arabidopsis* | [52] |
| c02291;c02292 | AtGolS3(Galactinol synthase 3) | At1g09350 | *Arabidopsis* | [52,53] |
| c22124 | zinc finger (C2H2 type) protein family | At1g14580 | *Arabidopsis* | [52] |
| c29180 | RAP2.1(AP2 domain DNA-binding protein) | At1g46768 | *Arabidopsis* | [52,53] |
| c01151 | serpin, putative | At1g47710 | *Arabidopsis* | [52] |
| c16244 | expressed protein | At1g48100 | *Arabidopsis* | [52] |
| c20722 | proline-rich protein | At1g51090 | *Arabidopsis* | [52,53] |
| c02177;c02183;c02184 | POT protein | At1g52190 | *Arabidopsis* | [52] |
| c09647;c34554 | putative POT protein | At1g69870 | *Arabidopsis* | [52] |
| c21276 | flavin-containing monooxygenase | At1g62570 | *Arabidopsis* | [52] |
| c26735 | vauolar cystein proteinase (beta-VPE) | At1g62710 | *Arabidopsis* | [52] |
| c16657;c31605 | alcohol dehydrogenase | At1g77120 | *Arabidopsis* | [52] |
| c31672 | Gamma-tyionins family | At2g02100 | *Arabidopsis* | [53] |
| c28638 | putative tetracycline transporter protein | At2g16990 | *Arabidopsis* | [52] |
| c04133 | AtGRP7(Glycine-rich RNA-binding protein) | At2g21660 | *Arabidopsis* | [53] |
| c07003;c07007;c08549;c22362; | putative GDSL-motif lipase/hydrolase | At2g24560 | *Arabidopsis* | [52] |
| c24405;c28307;c32945 |  |  |  |  |
| c32516 | AtOEP16 | At2g28900 | *Arabidopsis* | [52,53] |
| c28791 | calmodulin-like protein (TCH3) | At2g41100 | *Arabidopsis* | [52] |
| c09568 | cor15a(Late embryogenesis abundant protein) | At2g42540 | *Arabidopsis* | [52,53] |
| c33058 | putative endochitinase | At2g43620 | *Arabidopsis* | [52] |
| c27215;c27960;c29461 | beta-glucosidase (GLUC) | At3g03640 | *Arabidopsis* | [52] |
| c26713;c30623;c32106;c34066 | putative disease resistance protein | At3g05660 | *Arabidopsis* | [52] |
| c04229;c09764;c28697;c28755 | aspartyl protease family | At3g54400 | *Arabidopsis* | [52] |
| c28693;c29804;c30145;c35528 | beta-glucosidase-like protein | At3g62740 | *Arabidopsis* | [52] |
| c27941 | EARLI1(Early arabidopsis aluminum induced 1) | At4g12480 | *Arabidopsis* | [52,53] |
| c28644 | pEARLI 1-like protein | At4g12490 | *Arabidopsis* | [52] |
| c34695 | Unknown protein | At4g14000 | *Arabidopsis* | [53] |
| c24605;c27113;c32473;c33345 | cytochrome P450 family | At4g22710 | *Arabidopsis* | [52] |
| c17747 | zinc finger (CCCH type) protein family | At4g29190 | *Arabidopsis* | [52] |
| c30547;c34395 | unknown protein | At4g30650 | *Arabidopsis* | [52,53] |
| c31689 | cytochrome P450 (ATR4/CYP83B1) | At4g31500 | *Arabidopsis* | [52] |
| c01719 | Pdc1(Pyruvate decarboxylase-1) | At4g33070 | *Arabidopsis* | [52,53] |
| c18849;cg18850;c28246 | MYB family transcription factor (MYB32) | At4g34990 | *Arabidopsis* | [52] |
| c04688;c06528;c24107 | sugar transport protein | At4g35300 | *Arabidopsis* | [53] |
| c01329;c33575 | thaumatin-like protein | At4g36010 | *Arabidopsis* | [52] |
| c30346 | ATFP6(Farnesylated protein) | At4g38580 | *Arabidopsis* | [52,53] |
| c32480 | like ABA-responsive protein | At5g13200 | *Arabidopsis* | [52] |
| c02025;c29094 | sucrose synthase I (SUS1) | At5g20830 | *Arabidopsis* | [52] |
| c15913;c20019;c24343;c27574; | serine/threonine protein kinase-like | At5g25110 | *Arabidopsis* | [52] |
| c29104;c29417;c29521;c30986; |  |  |  |  |
| c31121;c32485;c32889;c34184 |  |  |  |  |
| c28437; |  |  |  |  |
| c15556;c16354;c27608 | protein phosphatase 2C (PP2C), putative | At5g27930 | *Arabidopsis* | [52] |
| c27111 | Ca2+-transporting ATPase (ACA8) | At5g57110 | *Arabidopsis* | [52] |
| c09990 | HvCBF3-Dt | AY785845 | Barley | [61] |
| c09993 | HvCBF8A-Dt | AY785868 | Barley | [61] |
| c09992 | HvCBF10A-Dt | AY785882 | Barley | [61] |
| c31655 | HvCBF12-Dt | DQ095157 | Barley | [61] |
| c15913;c19697;c26993;c32459 | OsDREB1C | AP001168 | Rice | [61] |
| c34162 | OsDREB1G | AP005775 | Rice | [61] |
| c18850;c33806 | MYB4(an R2R3-type MYB) | Y11414 | Rice | [62] |
| c28499 | TmCBF7 | AY785904 | Wheat | [61] |
| c22149 | TaCBF9 | AY785905 | Wheat | [61] |
